# Political Attitudes Develop Independently of Personality Traits

**DOI:** 10.1371/journal.pone.0118106

**Published:** 2015-03-03

**Authors:** Peter K. Hatemi, Brad Verhulst

**Affiliations:** 1 United States Studies Centre, University of Sydney, NSW, Australia; 2 Political Science, Pennsylvania State University, University Park, PA United States of America; 3 Virginia Institute for Psychiatric and Behavioral Genetics, Virginia Commonwealth University, Richmond, United States of America; University of Hong Kong, HONG KONG

## Abstract

The primary assumption within the recent personality and political orientations literature is that personality traits cause people to develop political attitudes. In contrast, research relying on traditional psychological and developmental theories suggests the relationship between most personality dimensions and political orientations are either not significant or weak. Research from behavioral genetics suggests the covariance between personality and political preferences is not causal, but due to a common, latent genetic factor that mutually influences both. The contradictory assumptions and findings from these research streams have yet to be resolved. This is in part due to the reliance on cross-sectional data and the lack of longitudinal genetically informative data. Here, using two independent longitudinal genetically informative samples, we examine the joint development of personality traits and attitude dimensions to explore the underlying causal mechanisms that drive the relationship between these features and provide a first step in resolving the causal question. We find change in personality over a ten-year period does not predict change in political attitudes, which does not support a causal relationship between personality traits and political attitudes as is frequently assumed. Rather, political attitudes are often more stable than the key personality traits assumed to be predicting them. Finally, the results from our genetic models find that no additional variance is accounted for by the causal pathway from personality traits to political attitudes. Our findings remain consistent with the original construction of the five-factor model of personality and developmental theories on attitude formation, but challenge recent work in this area.

## Introduction

Research exploring the relationship between personality traits and political attitudes has a long history [1–4], but a great deal of confusion about the nature of this relationship has recently surfaced. Early conceptions of personality viewed attitudes and personality traits as independent yet related, through genetic mechanisms [2,5,6]. This work was informed by developmental approaches that proposed that political preferences are an integral part of human development [7,8]. As personality theory and measurement progressed, political values were incorporated into various personality theories in several ways, including several of the dimensions and subfacets of the five-factor model (FFM) as well as Eysenck’s Big 3. For example, liberal and conservative values are explicitly used to define the Values facet of the Openness dimension [9] (S1 File). Tough versus Tendermindedness, originally a measure of political attitudes outlined by Eysenck [2], is embedded in the FFM trait of Conscientiousness [10,11], and moral and economic values are included in Agreeableness (see S2 File for some examples).

Research over the last decade has begun to depart from these original conceptions of personality, and view political attitudes as exogenous to personality measures. Specifically, the overwhelming majority of recent research hypothesizes a strict chain of causality between personality and political values, through explicit theorizing or implied through statistical models [12–19]. In this way, the same construct may reside on both sides of the predictive equation. Such a view, however, may not be entirely unjustified because modern personality dimensions, even in abbreviated forms, are intended to measure several sub-facets that do not explicitly include political attitudes. For example, in addition to political values, the Openness to Experience dimension measures openness to ideas, aesthetics, fantasy, feelings and actions, all of which are believed to capture the basic motivational states that make a person amenable to novel experiences, influencing their tolerance of ambiguity, and desire for cognitive closure, in order to reduce uncertainty in their environment. These motivational states, so it has been theorized, have an independent causal role in the development of liberal or conservative attitudes [20,21].

A third stream of research relying on behavioral genetic methods and developmental theories adds further complexity. These theories suggest that it is unlikely that genetic influences will operate directly on a complex trait. Rather, genetic influences are polygenetic and multifactorial. This approach suggests that although personality traits and attitudes are correlated, they are largely related through a third underlying latent genetic factor [5,22,23].

In summary, hundreds, if not thousands, of papers on personality and political traits have been published claiming causality, while some claim covariation, whereas the foundational literature considers political attitudes to be embedded within personality. The results from these separate research streams provide a considerable amount of empirical content, but are inconsistent in both empirical results and their theoretical underpinnings, resulting in an unclear picture of the relationship between personality and political attitudes. What has so far remained absent is a longitudinal exploration of the causal role of personality on political orientations. That is, a conventional test where changes in variable x (personality) result in a changes in variable y (political attitudes) over the long term, has yet to be presented. Here, we seek to take this next critical step in order to clarify the nature of the relationship between personality and attitudes, and integrate extant findings in this area. Using two independent longitudinal samples, we examine the joint development of personality traits and attitude dimensions to explore the underlying causal mechanisms that drive the relationship between the two and to resolve some of the theoretical inconsistencies in the current literature. Over a ten-year period, we find little evidence that changes in personality *cause* changes in political attitudes. Rather, whatever covariance is shared between personality traits and attitudes is present early in life, and is driven by a common set of dispositional influences, suggesting that the covariance they share are manifestations of the same underlying trait. The results are highly consistent with the original construction of the five-factor models of personality and developmental theories on attitude formation [9], as well as earlier constructions of personality [2,24]. In the absence of explicit measures on attitudes, personality measures provide some insight into one’s ideological disposition, because measures of personality traits were not designed to predict ideological attitudes, rather, they were designed to include them in a limited form [2,9–11,25].

### The Nature of Personality Traits and Political Attitudes

Personality traits have historically been viewed as innate dispositions that emerge early in life, are relatively stable, and generally guide behavior in a readily identifiable manner [26–29]. Political attitudes, on the other hand, were initially characterized as highly context dependent, viewed as responses to the immediate social environment [30,31], and believed to stabilize in a person’s late 20’s [32]. More recent conceptualizations of personality and attitude formation however, provide the foundation for an important modification of these views. Specifically, four questionable assumptions have exacerbated the belief that personality traits cause people to develop political attitudes: 1) personality traits are established early in life and they remain consistent across the lifespan [33,34]; 2) by extension, it is assumed that regardless of which specific dimension is being examined, personality traits are highly stable both temporally and contextually. For example, Costa Jr and McCrae [34], the architects of the NEO-PI-R, argue:

Personality is not a product of the life course … but a robust resilient set of dispositions within the individual that themselves help shape the life course. People are not mere pawns of the environment, but active agents who steadfastly pursue their own style of being throughout life.

Furthermore, 3) it is assumed that changes in personality over time are primarily a function of measurement error [35]. Borrowing heavily from this sentiment, the personality and politics literature largely portrays personality traits as almost perfectly stable across a person’s life and driven by genetic causes that then influence attitudes [12,17,18,36]. Finally, 4) the correlations between personality traits and political attitudes are discussed as if they capture a substantial amount, if not majority of the variation in attitudes.

None of these assumptions hold true. Empirical evidence undermines the assumption of near perfect stability and meta-analytic reviews demonstrate that personality continuity peaks much later in adulthood than is proffered based on the personality and attitudes literature [37,38]. Test-retest correlations of personality traits are significant and substantial but not overly large, whether in adulthood or from childhood to early adulthood [39–41]. This implies the magnitude of stability is not as high as “essentialists” claim, but too high to be entirely fluid. Personality stability increases with age, especially after the formative years of adolescence, and peaks after age 50. Yet, stability decreases as the time interval between observations increases, consistent with a gradual model of change rather than a critical event having a large discontinuous effect on the trait. This evidence runs counter to views that personality traits are fixed and unchanging, or that change is simply the result of measurement error, “facts” that are often used as the basis of the causal influence of personality on attitudes. In addition, contextual effects and critical life events influence personality development [42]. Malleability is typified by the particularly low test-retest correlations for personality measures during periods of physical, cognitive, and social development [43], and implies that the expression of personality traits varies greatly not just over time, but also in different situations at the same point in time. This perspective is consistent with a substantial body of personality research that suggest personality traits are contingent on the specific situation and even trivial situational differences can vastly decrease the observed relationship between the same personality traits within the same person [44,45]. As the situation becomes more discrepant both temporally and contextually, stability decreases.

The dynamic component of personality also appears to be trait specific. In a review of over 80 studies, Roberts et al. [40] find that Agreeableness and Conscientiousness increase in young adulthood and middle age, while Neuroticism decreases in adulthood [41]. Openness to Experience increases in adolescence, peaks in young adulthood and subsequently decreases with age. It is the least stable of the FFM traits. In total, people become more dominant, agreeable, conscientious, more emotionally stable and less open to experiences as they age. Even though one’s behavior is to some extent situation specific and changes over time, there is still individual consistency. Accordingly, personality traits reflect two separate factors. A stable component whereby a person’s personality predispositions affect contemporary personality expression, and a dynamic component that is updated or learned, whereby personality dispositions are functions of one’s response to the contemporary environment, the environment one selects into not guided by personality traits, and what has been learned since his or her personality was last assessed [46].

Finally, the majority of studies find the correlations between most personality traits and attitude dimensions (e.g., economic, social, religious, defense, etc.) are either not significant or the effect sizes are small making the substantive effects minor. For the personality traits and attitude dimensions that are correlated, the largest correlations typically range between 0.2 to 0.3 for the FFM traits and up to .4 for Eysenck’s traits [17,18,22,23]. For the FFM and Eysenck’s personality traits, positive correlations between Openness and social liberal attitudes, Neuroticism and economically liberal attitudes, Conscientiousness and socially conservative attitudes, the P-Scale and pro-military/defense attitudes, and Social Desirability and socially liberal attitudes are the most consistently found. The traits of Extraversion and Agreeableness are not consistently related to any political attitude dimension in direction or significance.

On the other hand, political attitudes are much more stable than is often presented in the personality and politics literature [47–50]. Indeed, challenges to the stability of personality traits are parallel to the assumption that attitudes are transitory, albeit in the opposite direction. Converse [31] proposed that political attitudes are randomly associated with other political attitudes, and overtime consistencies are dismally low. Poignantly, Achen [47] demonstrated that once correcting for measurement error, the attitudes that the public holds are, in fact, remarkably constrained. This view remains the most empirically supported [32,48–50], though Converse’s early work remains highly cited. In addition, consistent with research on the aging-stability hypothesis, attitudes are more stable as people age, with stability rates on par with personality [51–54]. While attitudes are responsive to contextual variation, the public has more constrained beliefs than typically assumed, akin to personality traits, and people become more consistent as they age. And similar to personality, the persuasion literature is replete with examples of attitude change based upon relatively subtle experimental manipulations, exposure to new information, and variation in an individual’s motivation and ability to think about information systematically [55,56]. Accordingly, while attitudes may be more stable than typically assumed they are not completely stable across time and vary in different contexts.

Thus, the general assumptions in the personality literature and the attitudes literature overstate the stability of personality traits and downplay the stability of political attitudes. When these literatures are integrated, as in the political psychology case, this culminates in the assumption that personality causes people to develop directional political attitudes.

Nevertheless, contrary to the recent assumption in the personality and politics literature, both an individual’s personality and their attitudes develop and change throughout life [32,37,38,48–50]. Moreover, when attitudes are assessed using stimuli that young children can cognitively comprehend, such as measures based upon picture categorization tasks or cognitive interviews, attitudes can be measured effectively during the same developmental period as personality temperaments [57–62]. Furthermore, before the age of 15 the structure of children’s attitudes begins to resemble that of adults [63–66]. Thus, it appears that personality traits and political attitudes are formed, changed and maintained during the same developmental stages and one is not subsequent to the other.

Until recently, determining the causal direction and including genetic influences remained both statistically difficult, theoretically problematic, and generally impractical as there has been a lack of data on the subject. However, the incorporation of behavioral genetic studies provides a bridge to address this relationship. Both personality traits and attitudes are influenced by genetic factors [67–70]. A series of recent studies found that the majority of the covariation between personality traits and political attitudes is primarily a function of shared additive genetic covariation [22,23,71]. Yet, the covariation between the additive genetic components of personality traits and attitudes accounts for a relatively small fraction of the total genetic variance in these dimensions [5]. Thus, the majority of the additive genetic variation in both attitudes and personality traits is not shared with each other. Speaking directly to the question of causality, using a direction of causation model that relies on genetic differences in the covariance structure, Verhulst et al. [23] found that rather than personality traits *causing* people to develop directional attitudes, covariation between the two is largely due to common genetic influences (for a discussion of the method see [72]). Such a view is consistent with genetic theory: specific genes for a given political attitude or personality trait do not exist [73–75]. Rather, genes encode proteins that execute a series of physiological mechanisms that influence emotive and cognitive states, which influence perceptions, behaviors, personality traits and attitudes in reaction to environmental stimuli; all of these forces operate differentially through different life stages. The same set of genes in combination with a plethora of different environments result in myriad distinct behavioral phenotypes including the two domains relevant to the current discussion, political attitudes and personality traits.

These findings required a critical revision of the causal assumption. That is, the assumption that personality is causing attitudes, or that the genetic factors that influence personality cause the genetic factors that influence attitudes, appears unlikely. Rather, a latent genetic factor appears responsible for the covariation between, and joint development of, personality and political attitudes [22]. While this literature suggests that personality traits do not cause people to develop attitudes, two critical elements remain unaddressed. First, these studies did not include the personality trait most proffered to cause political attitudes: Openness to Experience [17,18,25]. Second, one imperative exploration remains in order to test whether personality traits and political attitudes develop in tandem rather than sequentially: a longitudinal study of personality and politics in a genetically informed sample. We conduct such an exploration here. If personality traits cause people to develop liberal or conservative political attitudes, changes in personality would be associated with changes in political attitudes, but not necessarily the reverse. Such a test is critical because the lack of a causal link from personality to politics would require a major revision of the current literature, which is critically dependent upon this thesis.

Accordingly, we proceed by exploring three empirical questions: (1) how stable are personality traits and attitudes over time, (2) what is the relationship between personality traits and political attitudes across time and (3) what are the sources of stability and change within the attitudinal dimensions?

## Materials and Methods

The structure of the data used in this paper is central to several of the hypotheses that we test and the subsequent conclusions we draw from our results. In the current study we analyze two separate longitudinal data sets, each with two waves of data collection approximately 10 years apart: a Mature Adult Cohort (Adult) and an Adolescent to Adult Cohort (Adolescent). We focus on the combination of personality traits and attitude dimensions identified in the extant literature that share the largest covariance, our data limitations withstanding. In the Adult cohort these include the relationship between attitudes and Eysenck’s P and Social Desirability [22,23]. In the Adolescent Cohort we focus on Openness to Experience and social attitudes. Due to the lack of substantial correlations between most personality traits and attitude dimensions, and limitations in the data, relationships between other personality and attitude dimensions are explored in a more limited fashion or not at all. In this regards, it is important to highlight the unique nature of this data. Only a data set in which longitudinal measures of both personality and attitudes, in a genetically informed sample, can begin to answer the important question of causality. Data collection and analysis was approved and conducted within the guidelines set forth by the Human Research Ethics Committee of the Queensland Institute of Medical Research. Written informed consent was obtained from each participant (and for those aged <18, their parent or guardian) prior to phenotype collection.

### Participants

In the Adult Cohort, the first wave of data collection (1980) consisted of 7,610 Australian twins and siblings, aged 19–78 (μ = 33, σ = 14), who were assessed by the Queensland Institute for Medical Research as part of a study exploring a wide range of health, cognitive and behavioral traits [69,76]. Approximately 10 years later 5,904 of these individuals were re-ascertained for a follow-up study. Almost half the sample changed in marital status, had children, and changed their occupation or educational status between measurements speaking directly to the profound changes in life circumstances across different stages of adulthood. Details on the data collection techniques, response rate and other characteristics are specified in previously published studies [68,77–79].

The Adolescent Cohort is comprised of data collected as part of a longitudinal study of cognition in adolescent twins and their siblings [80,81]. Data from 1,061 twins and siblings was collected in 1998, when subjects were approximately 16–19 years old. Approximately 10 years later, between 2008–2009, 300 twin pairs were re-ascertained for personality and attitudes as part of a follow-up study.

### Measures

The Adult Cohort was assessed using Eysenck’s Big 3 personality inventory. As mentioned above, we focus on Eysenck’s Psychoticism (P), a blend of tough-mindedness, risk-taking, sensation-seeking, impulsivity, and authoritarianism) and Social Desirability (SD). Personality factors were coded so that higher values reflect more of the trait. Because the same personality items were asked in both the first and second waves of the Adult study, examination of the mean shifts in personality as people age is possible. To do so, we constrain the factor loadings and thresholds for all of the items to be equivalent and allow the disturbances of the identically worded items to correlate across measurement occasions. For the political attitudes, we constructed two attitudinal dimensions drawn from a 28-attitude item Wilson and Patterson [82] political attitude questionnaire. Again, questions were worded exactly the same in the 1980 and 1990 assessments. Attitude factors were coded so that higher values reflect more conservative attitude positions. The two attitudes dimensions, social and religious ideology (Table 1), remain highly salient in the recent literature. We required attitude dimensions that could be constructed in both waves in both samples in order to properly model the causal relationship. Unfortunately, there were not enough items available to construct additional attitudinal dimensions (i.e., economic) with adequate statistical properties across waves. For example, several of the items did not load onto any standard political attitude dimensions (e.g., pajama parties) and other items did not cluster together into meaningful and interpretable factors and had unacceptable levels of model fit to be considered for analysis, such as a RMSEA > .10. For this reason we did not conduct analyses on the relationship between neuroticism and economic attitudes, also identified in the literature [17,22].

**Table 1:** Unstandardized CFA Loadings for the Attitudinal Factors in the Adult Study.

	Social Ideology	Religious Attitudes
Abortion	-0.882 (0.011)	
Divorce	-0.690 (0.011)	
Birth Control	-0.582 (0.019)	
Working Mothers	-0.375 (0.015)	
Bible Truth		0.820 (0.007)
Church Attendance		0.739 (0.008)
Divine Law		0.723 (0.008)
Sabbath Observance		0.698 (0.009)
Chastity		0.527 (0.011)
Evolution		-0.497 (0.012)
Censorship		0.420 (0.012)

Note: These factor loadings are taken from the final model for the adult study presented in Fig. 1 (standard errors of the factor loadings are in parentheses). Although the specific loadings vary for each model that is estimated, this variation is minimal. All of the factor loadings are significant beyond the p < .001 level. To equate the models across time, the loadings and thresholds are constrained to be equal across measurement occasions. The disturbances were clustered by family to account for the non-independence of the observations. To ensure that the relationships between the latent factors were not inflated by correlated disturbances at the item level, we specified covariances between items with the exact same wording across measurement occasions.

**Fig 1:**
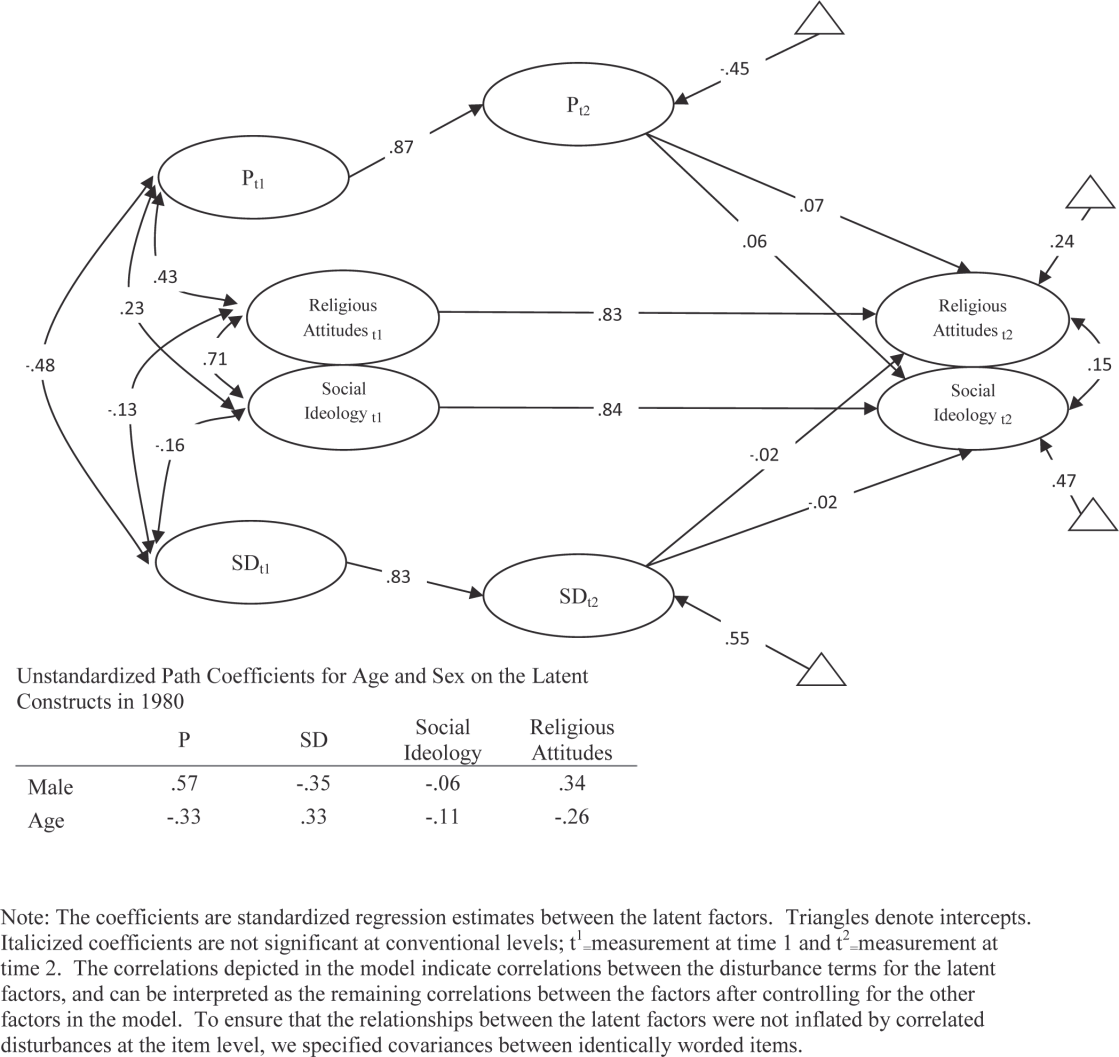
The Longitudinal Relationships between Psychoticism, Social Desirability, Social Ideology Attitudes and Religious Attitudes in the Adult Study.

To ensure consistency across time, the attitudinal factor structure in 1980 and 1990 were equated just as the personality items were equated. The models that we estimate have a large number of parameters; we present the factor loadings for the latent personality factors in Table 2 and the factor loadings for the latent attitudinal factors are in Table 1, and a path diagram for the full structural model in Fig. 1. The Root Mean Square Error of Approximation (RMSEA) for this model is 0.028, the TLI is 0.954 and the CFI is 0.928, indicating that the model is a very good fit to the data (χ^2^ = 4620.40).

**Table 2:** Unstandardized CFA Loadings for the Eysenck Personality Traits in the Adult Study.

	Psychoticism	Social Desirability
Would you take drugs which may have strange or dangerous effects?	0.753 (0.038)	
Do good manners and cleanliness matter much to you?	-0.51 (0.033)	
Would you like other people to be afraid of you?	0.323 (0.037)	
Do you stop to think things over before doing anything?	-0.246 (0.024)	
Do you try not to be rude to people?	-0.191 (0.027)	
Do you think people spend too much time safeguarding their future with savings and insurances?	0.124 (0.021)	
Have you ever taken advantage of someone?		-0.700 (0.010)
Have you ever said anything bad or nasty about anyone?		-0.656 (0.014)
Have you ever taken anything that belonged to someone else?		-0.631 (0.011)
Have you ever cheated at a game?		-0.625 (0.011)
Were you every greedy by helping yourself to more than your share?		-0.616 (0.011)
Do you always practice what you preach?		0.570 (0.011)
Are all your habits good and desirable ones?		0.540 (0.012)
Have you ever broken or lost something belonging to someone else?		-0.527 (0.012)
Have you ever blamed someone for what was really your fault?		-0.523 (0.012)
As a child, were you ever ‘fresh’ towards your parents?		-0.429 (0.013)
If you say you will do something, do you always keep your promise?		0.419 (0.014)
Do you sometimes put off until tomorrow what you ought to do today?		-0.383 (0.016)

Note: Factor loadings are from the final model for the adult study presented in Figs. 1–2 (standard errors of the factor loadings are in parentheses). Although the specific loadings vary for each model estimated, variation is negligible. All of the factor loadings are significant beyond the .001 level. To equate the models across time, the loadings and thresholds are constrained to be equal across measurement occasions. Disturbances were clustered by family to account for the non-independence of the observations. We specified covariances between items with the exact same wording across measurement occasions to ensure that the relationships between the latent factors were not inflated by correlated disturbances at the item level.

In the Adolescent cohort we focus on the relationship between Openness to Experience and Social Ideology, also measured by a Wilson-Patterson attitude index. During the first assessment of the Adolescent study, personality traits were measured by a modified version (Australian) of the full 244 item NEO PI-R (Form S) with complete subscales for each of the primary dimensions. When the respondents were reassessed as young adults ten years later, the personality dimensions were measured by a truncated 44-item FFM scale [83], which had become a standard measure. To solve the problem of differential reliability between scales, we matched the items from the full scale to those in the short scale. The factor structures for Openness to Experience in both scales are presented in Table 3. Some items are slightly different between the scales, but consistent with previous comparisons [84]. The full and the reduced scales tap into the same constructs, and are compatible measures. Because the shortened scale was used in the second assessment we can only assess the consistency between the latent constructs and not the mean differences between the specific items or observed levels of the traits. The RMSEA for this model is 0.041, again indicating that the model explains the data very well. The TLI is .79 and the CFI is .80, which is expected as both are known to be lower in complex structural models (χ^2^ = 1342.83). Recall, we did not explore the longitudinal relationship with other personality factors in the Adolescent study because we did not have suitable measures at time 1.

**Table 3:** Unstandardized Factor loadings for the Openness to Experience in the Adolescent Study.

	Adolescence	Young Adults
I sometimes lose interest when people talk about very abstract, theoretical matters	-0.659 (0.041)	
Sometimes when I am reading poetry or looking at a work of art, I feel a chill or wave of excitement	0.609 (0.051)	
I often enjoy playing with theories or abstract ideas	0.606 (0.040)	
I have a wide range of intellectual interests	0.589 (0.038)	
I have a lot of intellectual curiosity	0.547 (0.039)	
Aesthetic and artistic concerns aren't very important to me	-0.421 (0.053)	
Watching ballet or modern dance bores me	-0.386 (0.060)	
I have a very active imagination	0.369 (0.042)	
I'm pretty set in my ways	-0.171 (0.042)	
is inventive.		0.572 (0.044)
is sophisticated in art, music, or literature.		0.556 (0.051)
likes to reflect and play with ideas.		0.553 (0.037)
values artistic, aesthetic experiences.		0.523 (0.043)
is original, comes up with new ideas.		0.517 (0.045)
has an active imagination.		0.516 (0.038)
is ingenious, a deep thinker.		0.476 (0.036)
is curious about many different things.		0.404 (0.037)
has few artistic interests.		-0.164 (0.050)

Note: These factor loadings are taken from the final model for the adolescent study presented in Fig. 3 (standard errors of the factor loadings are in parentheses). Although the specific loadings vary for each model that is estimated, this variation is negligible. All of the factor loadings are significant beyond the p < .001 level. The disturbances were clustered by family to account for the non-independence of the observations.

The measurement of political attitudes in the *Adolescent* study differs from the attitudinal measures used in the *Adult* study. Specifically, the political attitudes included at time 1 (age 16–19) were measured from the political values facet. As McCrae and Terracciano [10] note, Values facets “are considered attitudinal facet scales.” In the full NEO-PI-R, the Political Values dimension taps into moral values and social-political attitudes, and includes items like “I believe that the "new morality" of permissiveness is no morality at all”, “I consider myself broad-minded and tolerant of other people’s lifestyles.” “I believe that different ideas of right and wrong that people in other societies have may be valid for them”, “I believe letting students hear controversial speakers can only confuse and mislead them”, and “I believe that laws and social policies should change to reflect the needs of a changing world”. Thus the underlying latent construct that the values items are tapping into was designed to capture the same as the latent construct that the social ideological attitudes are capturing. The factor loadings on the latent factors are presented in Table 4. The second wave of the adolescent sample included 28 attitudes. However, In order to be consistent with the political values dimension, we included only those attitudes that captured social/moral attitudes.

**Table 4:** Unstandardized Factor loadings for the social political values in the Adolescent Study.

	Political Values	Social Ideology
I believe that loyalty to one's ideals and principles is more important than "openmindedness"	0.503 (0.045)	
I believe letting students hear controversial speakers can only confuse and mislead them	0.463 (0.046)	
I think that if people don't know what they believe in by the time they are 25, there's something wrong with them	0.396 (0.052)	
I believe that the different ideas of right and wrong that people in other societies have may be valid for them	-0.384 (0.049)	
I believe we should look to our religious authorities for decisions on moral issues	0.341 (0.057)	
I consider myself broadminded and tolerant of other people's lifestyles	-0.326 (0.036)	
I believe that laws and social policies should change to reflect the needs of a changing world	-0.272 (0.036)	
I believe that the "new morality" of permissiveness is no morality at all	0.179 (0.041)	
Abortion		0.477 (0.042)
Euthanasia		0.411 (0.037)
Evolution		0.391 (0.038)
Gay Marriage		0.374 (0.037)
Living Together		0.354 (0.041)
Government Funded Abortion		0.299 (0.038)
Medical Research		0.261 (0.034)

Note: These factor loadings are taken from the final model for the adolescent study presented in Fig. 3(standard errors of the factor loadings are in parentheses). Although the specific loadings vary for each model that is estimated, this variation is negligible. All of the factor loadings are significant beyond the p < .001 level. The disturbances were clustered by family to account for the non-independence of the observations.

## Results

### The Stability of Personality Traits over Time

The Structural Equation Model (SEM) of the longitudinal relationships between the personality traits and the political attitudes dimensions was conducted in Mplus 5.21 & 6 [85]. The stability of personality traits is central to the understanding of the relationship between personality and political attitudes. Based on the literature, we expect there should be a reasonable amount of stability with some change occurring over time. The stability coefficients, presented in Table 5, provide evidence that supports both stability and change in all of the personality traits. Our detailed analysis focuses on the P and SD dimensions in the Adult cohort and the Openness to Experience factor in the Adolescent cohort, but we include all of the possible Eysenck and FFM personality dimensions in the stability analysis. As can be seen in Table 5, in no case is a personality trait perfectly stable, but in all cases the personality traits are significantly related over time and there is considerable heterogeneity within the stability estimates.

**Table 5:** The Stability of Personality Across Time.

	Personality Dimension	Stability Coefficient	Intercept
Adult Study	Eysenck’s P	0.796 (0.048)	-0.406 (0.037)
	Extraversion (E)	0.775 (0.008)	-0.062 (0.011)
	Neuroticism (N)	0.729 (0.009)	-0.129 (0.012)
	Social Desirability (SD)	0.789 (0.010)	0.357 (0.013)
Adolescent Study	Openness to Experience	0.527 (0.064)	-
	Conscientiousness	0.740 (0.045)	-
	Extraversion	0.725 (0.046)	-
	Agreeableness	0.558 (0.067)	-
	Neuroticism	0.741 (0.047)	-

Note: The stability coefficients are asymmetric paths from a structural equation model between the latent personality factors at time 1 and time 2 (the standard errors of the stability coefficients are in parentheses). The Coefficients are standardized path coefficients between the latent factors to increase comparability between variables with potentially different variances. All models were estimated separately. For every model, the disturbances were clustered by family to account for the non-independence of the observations. To ensure that the relationships between the latent factors were not inflated by correlated disturbances at the item level, we specified covariances between identically worded items. In the adult study the fit statistics are as follows: P(RMSEA = .01, CFI = .99, TLI = .99, χ^2^ = 98.46); Social Desirability(RMSEA = .04, CFI = .95, TLI = .94, χ^2^ = 3283.90); Extraversion (RMSEA = .06, CFI = .94, TLI = .94, χ^2^ = 9915.81); Neuroticism (RMSEA = .06, CFI = .92, TLI = .92, χ^2^ = 7174.18). In the adolescent study the fit statistics are as follows: Openness (RMSEA = .06, CFI = .81, TLI = .78, χ^2^ = 598.76); Conscientiousness(RMSEA = .05, CFI = .89, TLI = .87, χ^2^ = 446.93); Extraversion(RMSEA = .06, CFI = .84, TLI = .81, χ^2^ = 551.71); Agreeableness(RMSEA = .04, CFI = .85, TLI = .83, χ^2^ = 398.30); Neuroticism(RMSEA = .07, CFI = .80, TLI = .77, χ^2^ = 563.63).

Neuroticism (N), Extraversion (E), Social Desirability (SD) and the P-Scale (P) are quite stable over time, across samples and personality measures. In the adolescent study there are notable differences between the stability of the personality dimensions. This is not unexpected, as personality traits become more consistent with age [40,86], and it is likely that the adult study captures this greater stability. Openness to Experience is the least stable trait (stability coefficient = 0.527). This finding is consistent with previous studies, as the component facets of Openness are the most loosely related of any of the five factors, and thus the weakest in replication studies [10]. Because exactly the same personality instrument was used in both measurement occasions in the Adult Cohort, we were able to assess population-level shifts over time. For P, E and N, as people aged they expressed lower levels of the trait. Alternatively, aging increased the respondents’ level of SD. These shifts in the intercepts suggest significant directional changes in personality occur during adulthood. Therefore, in both the adult and adolescent studies there is a significant level of change that occurs within an individual’s personality, and also a reasonable level of consistency across time.

### The Stability of Attitudes Across Time

Using the same analytical procedure, it is also possible to gain some traction on the question of attitude stability. As can be seen in Table 6, the stability of political attitudes is greater or equal to the stability of the related personality dimensions, even though the constructs were measured ten years apart and numerous personal, cultural and political shifts occurred during these time periods.

**Table 6:** The Stability of Attitudes Across Time.

	Attitude Dimension	Stability Coefficient	Intercept
Adolescent Sample	Social Ideology	0.515 (0.097)	-
Adult Sample	Religious Attitudes	0.837 (0.008)	0.095 (0.012)
	Social Ideology	0.817 (0.014)	0.132 (0.016)

Notes: The stability coefficients are standardized regression estimates between the latent factors (the standard errors of the stability coefficients are in parentheses). Models were estimated separately. The disturbances were clustered by family to account for the non-independence of the observations. The RMSEA is 0.027 (CFI = .99, TLI = .99, χ^2^ = 576.95) for the Religious attitudes model, 0.054(CFI = .97, TLI = .96, χ^2^ = 581.21) for the Social Ideology model and 0.038 for the adolescent social ideology model (CFI = .89, TLI = .87, χ^2^ = 222.36). The R^2^ for religious attitudes is 0.701, Social Ideology is 0.667, 0.265 and for the adolescent social ideology model. To ensure that the relationships between the latent factors were not inflated by correlated disturbances at the item level, we specified covariances between identically worded items.

Again, the results from the Adolescent cohort are broadly consistent with the results for Adult cohort. Moreover, the same interpretation of this increased stability apply to the attitudes measured in the Adult cohort as applied to the personality traits, and because the scale is exactly the same in both waves of the Adult cohort, measurement issues are minimized. Furthermore, previous literature suggests that older adults have more stable attitudes than younger adults [30,50]. Importantly, with respect to mean trends over time in the Adult study, and consistent with the literature on age and ideology, as people age, they profess slightly more conservative attitudes.

### The Relationship Between Personality Traits and Political Attitudes Over Time

Few studies take into account the developmental nature of both political attitude dimensions and personality traits. This is important because at the same time personalities are developing, people are also forming their attitudes that presumably guide their political preferences [58,63]. Reconsidering the causal role of personality on attitudes leaves the door open for the possible pathway of simultaneous development of personalities and political preferences.

Using longitudinal data it becomes possible to test the assumption that personality causally influences political preferences. Accordingly, if a causal relationship does exist, we would expect personality traits at time 1 to predict personality traits at time 2, and time 2 personality should mediate the effect of time 1 personality on time 2 political attitudes. Simply put, *if personality traits cause political preferences, changes in personality should correspond with changes in political attitudes*. Alternatively, if a causal structure does not exist, personality traits and attitudes should remain fairly stable or fluctuate independently across time and adult personality traits should have little influence over adult attitudes after controlling for previously measured personality and attitudinal measures.

To test these competing hypotheses we estimate two structural equation models for each cohort using the personality and attitudinal measures discussed previously. In the first model for each cohort, we restrict our analyses to second measurement occasion and specify a causal relationship flowing from the personality traits to the attitudes, which corresponds with the dominant approach in the literature where conclusions are drawn from cross-sectional data. This is a baseline model and we present it to remain consistent with the existing literature. In the second model, we incorporate prior attitudes and personality traits into the equation to more adequately assess causality. Specifically, this model allows us to test whether changes in personality cause corresponding changes in attitudes, which is the essence of the causal structure implied by the personality and politics literature.

**Fig 2:**
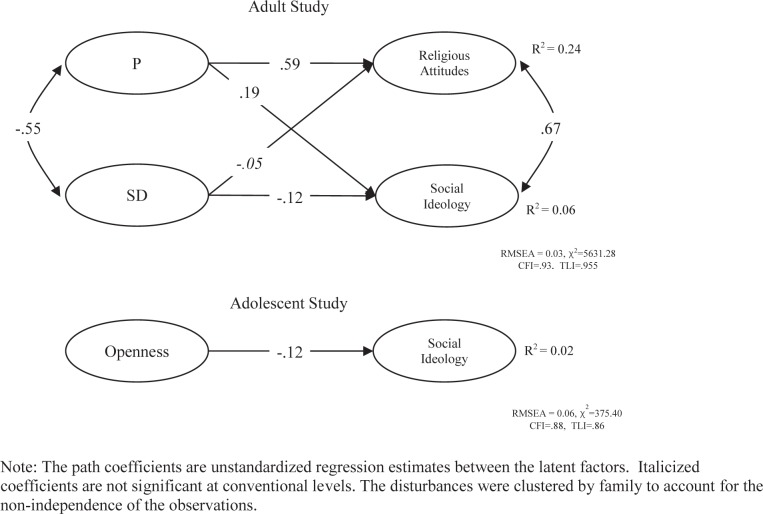
The Cross-Sectional Relationship between Personality and Social Attitudes at Time 2 only.

In Fig. 2, we present the models examining only the second measurement occasion estimated for the personality to attitudes causal structure for both studies. In the top panel we present the adult data where both P and SD predict the Social Ideology attitude factor in the same way that Openness to Experience predicts Social Ideology. The effect of P on the political attitudes is quite strong for Religious attitudes while noticeably weaker for Social Ideology. Alternatively, the effect of Social Desirability is not significant for Religious attitudes and statistically significant though relatively weak for Social Ideology. As can be seen in the bottom panel of Fig. 2, when we restrict the analysis to the second measurement occasion in the Adolescent cohort, higher levels of Openness to Experience in young adults do not meaningfully predict the endorsement of liberal political attitudes. The relationship is significant but inconsequential. Importantly, conclusions based only on data from the second wave would conclude that personality traits might have a meager causal role in developing political attitudes.

The full longitudinal model for the Adult Study is presented in Fig. 1. What becomes immediately apparent is that controlling for prior attitudes substantially reduces or eliminates the impact of personality on political attitudes. It is important to note that the paths from personality traits to the attitudes have a slightly different interpretation than in the previous figure. Because of the autoregressive components in personality traits and political attitudes in this model, the interpretation of the coefficients are based on changes in the constructs over time. Thus, a one standard deviation increase in the respondent’s level of P between 1980 and 1990 corresponds with being 0.072 standard deviations more conservative in Religious Attitudes and 0.066 more conservative in Social Ideology. Importantly, the substantial relationship between P and both attitude dimensions in 1980 is strongly attenuated by 1990. Furthermore, changes in the mean levels of the central dimensions also tell a very interesting story. On average respondents decreased in their levels on P, while increasing in both religious and social conservatism. *As such, the observed changes in the means of P and the political attitudes were in the opposite direction from what would be expected if changes in personality caused changes in political attitudes*.

Similarly, the changes in the respondent’s level of Social Desirability over this time period are unrelated to changes in the attitudinal dimensions. Seemingly paradoxically, and apparently consistent with the assumption of causality, this suggests that changes in P over time correspond with changes in political attitudes in the expected direction. Thus, the means that at the population level P and the political attitude dimensions are moving in the opposite direction of what would be expected, but a very minor correlation between P and the attitudes, resulting in a slightly positive regression coefficient, as a function of heterogeneity in the trajectories within the population, or random effects. This change, however, is far too small to support the assumption that personality traits play a significant causal role in the development of attitudes. The causal role, at least over a ten-year period, is unlikely and at best minimal.

**Fig 3:**
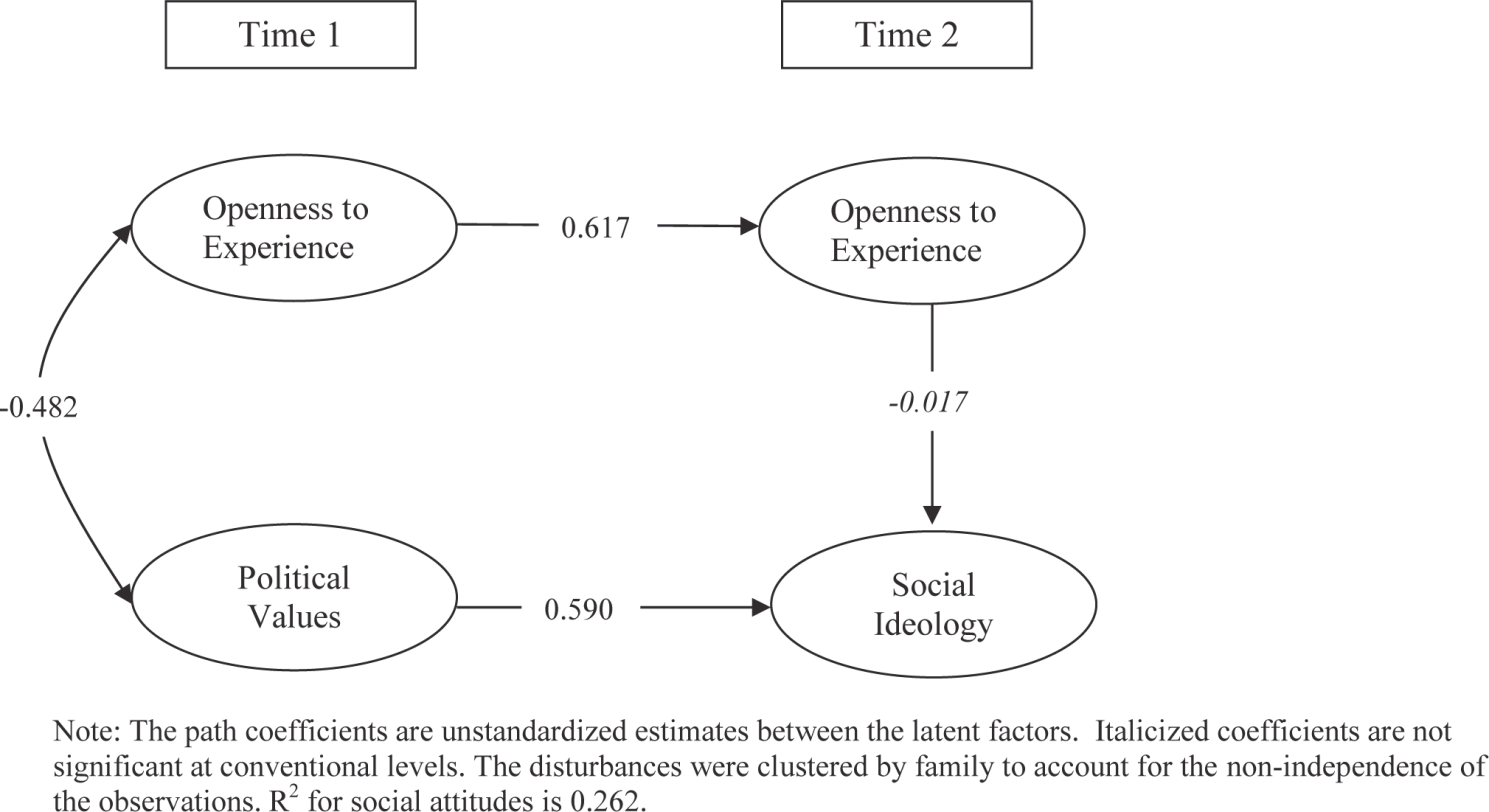
The Longitudinal Relationship between Openness to Experience and Social Attitudes in the Adolescent Study.

The model presented in Fig. 3 depicts the longitudinal relationship between Openness to Experience and political attitudes in the adolescent sample. Openness to Experience and political values in adolescence are strong predictors of Openness to Experience and Social Ideology as young adults, respectively, with the caveat that there is more change occurring within the traits over time relative to the adult sample. More interesting, the path from Openness to Experience to social ideological preference in young adults is *not* statistically significant. Therefore, changes in the respondents’ level of Openness to Experience between adolescence and young adulthood are unrelated to changes in political values over the same period. Thus, it appears that the predictive relationship between Openness to Experience and political values as young adults is simply an attenuated expression of the correlation between the two traits earlier in life.

Both samples include twins, allowing us to examine the latent sources of stability and change within the attitudinal dimensions. To do this we decompose the residual variances of the attitudes in the second stage of the analysis in order to test whether the variance in the attitudinal dimension is being captured at the genetic or environmental levels using OpenMx [87]. The three panels of Table 7 correspond with the three attitudinal dimensions explored. In Table 7, for each attitude, the total variance row denotes a simple variance decomposition irrespective of the predictors, the second row denotes the residual variance decomposition after extracting the Autoregressive (AR) Variance from the attitudinal dimension and the third row accounts for the additional reduction in residual variance after accounting for the path from personality at time 2 to the attitudinal dimension at time 2.

**Table 7:** Unstandardized residual variance in attitudes accounted for by prior attitudes and personality traits.

		Additive Genetic	Shared Environment	Unique Environment
Religious Attitudes	Total Variance	.48 (.41, .53)	.54 (.49, .58)	.51 (.50, .53)
	Residual Variance after AR	.12 (.06, .14)	.01 (-.09, .09)	.28 (.27, .29)
	Residual Variance after AR and Personality	.10 (.04, .12)	.00 (-.09, .09)	.26 (.26, .27)
Social Ideology Adults	Total Variance	.43 (.38, .48)	.45 (.40, .49)	.45 (.44, .47)
	Residual Variance after AR	.11 (.06, .13)	.05 (-.02, .09)	.21 (.21, .22)
	Residual Variance after AR and Personality	.10 (.05, .12)	.04 (-.04, .08)	.21 (.21, .22)
Social Ideology Adolescents	Total Variance	.53 (.35, .72)	.31 (.07, .57)	.46 (.39, .53)
	Residual Variance after AR	.21 (-.04, .48)	.00 (-.84, .84)	.41 (.34, .47)
	Residual Variance after AR and Personality	.21 (-.04, .47)	.00 (-.85, .85)	.41 (.35, .47)

Note: Confidence intervals are in parentheses. Fit statistics are presented in the text.

As can be seen in the Total Variance rows in each panel, for all three attitudinal dimensions there is a sizable amount of variance attributed to the additive genetic, shared and unique environmental variance components. After controlling for the AR component, all of these variance components decrease markedly and significantly. Specifically, for all three attitude dimensions, the shared environmental variance component decreases from statistically and substantively significant to meager and not significant (p > .05). Meanwhile, the additive genetic variance components decrease markedly but remain statistically significant in 2 models. Finally, while there is a substantively significant drop in the variance accounted for by the unique environment, this variance component comes to dominate the residual variance in the attitudinal dimensions. When the variance from the personality traits is then extracted from the variance components (the final row of each column), the variance components remain statistically unchanged, *underscoring the independence of the personality and attitudinal dimensions after accounting for the autoregressive effects of the attitudes and confirming what Eaves and Eysenck [5] suspected almost 40 years ago*; “Although we claim to show some common basis for differences in personality and attitude, we stress that, for the scales in question, the greater part of the genetic and environmental variation is specific to particular traits”.

Therefore, it appears that the autoregressive effects are accounting for virtually all of the common environmental variance, approximately 3/4 of the genetic variance and just over half of the unique environmental variance while *essentially no additional variance in the attitudinal factors is accounted for by the causal pathway from personality traits to political attitudes*. This leads to the conclusion that political attitudes are independent of personality traits and that changes in attitudes are primarily a function of changes in the environment or one’s unique experiences, and equally likely a function of differential gene expression at different stages of the life cycle, though we can only speculate on the later given our current data. Accordingly, attitudinal stability is affected by stable genes as well as stable environments. In both the Adult and Adolescent samples, the findings suggest that both constructs develop early in life, and this early covariance drives the relationships at later stages in life.

While we feel that the full longitudinal analysis presented above accurately captures the underlying theoretical model we are testing, another common method for testing causality is the cross-lagged panel design [88]. This model has been best described as a model for testing spurious relationships with some limitations [89]. We conducted Cross-Lagged correlation analyses as well (S3 File, S4 File, and S5 File). The interpretation of the results remains consistent with the structural models. That is, the Cross-Lagged model suggests that the relationship between the personality dimensions and the attitude dimensions is largely spurious.

### Limitations

As we clarify through each step of the analyses, the data provide many unique benefits, and as far as we know represent possibly the only data available to study the relationship between personality and political attitudes over an extended period of time, while modeling genetic and environmental influences of both stability and change. The data contain scales with a large number of items for personality measures, which have proven superior to the use of 10 item scales, and inclusion of the same measures of attitudes in two waves 10 years apart is highly informative and unique. Nevertheless, the data, as in any empirical study, remain fallible. In the Adolescent sample, the attitude measures at time 1 are not the same type as measured in time 2; recall at time 1 they rely on a values dimension. In addition, matching the 244 item NEO personality measure for Openness in wave 1 with the reduced 44 item measure in wave 2 may contribute to the lower stability estimated for Openness to Experience, though other studies that used the exact same measures in each wave returned similar findings [90]. In addition, we did not have the data to explore the longitudinal relationship between all personality traits and attitude dimensions identified in the literature (e.g., Neuroticism and economic attitudes). Finally, the data are not random, as is the nature of family samples, though our two different population-based samples support the same general conclusion.

## Discussion

There are four major interrelated findings from these analyses. First, while personality traits are fairly stable, they do change across the lifespan. Second, political preferences change over time, though they are at least, if not more, stable than many personality traits. Third, changes in personality traits and political attitudes over a ten-year period are scarcely related, if they are related at all. Fourth, whatever covariance is shared between personality traits and political attitudes is a manifestation of the same underlying genetic influence. Accordingly, the precursors of both of these constructs are established early in life but persist relatively independently into young adulthood. Importantly, they do not change in tandem as would be expected if a causal structure exists. Thus, we conclude that both personality traits and political attitudes are independently part of one’s psychological architecture (also see [23,91]),

These findings are consistent with the original development of personality theory, and fills in some of the gaps in the literature. Our findings also pose significant challenges to causal claims between personality and attitudes. Rather than demonstrating that a person’s personality traits cause them to develop directional ideological preferences, these results suggest that personality traits and attitudes develop in parallel. The relationship between attitudes and the personality traits that exists relatively early in life is substantively important only when we do not control for prior attitudes. When we appropriately control for prior measures of personality and attitudes, the relationship between personality traits and political values virtually disappears.

One may argue that the relationship between personality traits and attitudes at the first measurement occasion should be specified as causal. Accordingly, this would suggest that even though there is not a significant causal relationship from Openness to Experience as adults, a causal structure exists in one’s teens as Openness to Experience predicts the contemporary measure of political values. This specification, however, would require a substantial and radical revision of personality theory, to which no evidence exists. That is, it would mean personality stops working on behaviors or traits in adulthood.

Such a view, however, is not supported by the findings in our current model, nor by existing research, which finds the precursors to attitudes emerge as early as preschool [57,58,60]. Even if this alternative model is specified, the substantive relationship between the personality traits and political attitudes does not change. Most notably, it is highly probable that if we analyzed political attitudes and personality traits even earlier in life than we currently do, say in childhood or infancy, we would find the same pattern of relationships in adolescence as we do in the current model for young adults [33]. In this way, two related possibilities exist, that may obscure hypothesized effects. On the one hand, if this “new” initial wave of the study was collected before the respondents adequately developed the comprehension necessary to reliably respond to attitudinal or personality measures, no relationships would be found between the constructs. This would not necessarily be an indication that the relationship does not exist, but rather the measurement error is simply too great to provide an acceptable level of confidence in the relationship. In a related vein, if the relationship does exist in childhood, but only one measure can be reliably administered, one might falsely conclude that the more reliable measure was the cause of the relationship, when in reality, the effect of the immeasurable variable is observed through the other. For example, personality dispositions can be assessed from observations made by parents or teachers prior to the cognitive abilities necessary for political attitudes to be observed by these same raters. The same can be said for political dispositions, such as sharing, taking turns, equality and equity, but rarely are such measures assessed. Thus, it is tempting to assert that personality traits cause political attitude when what is really going on is omitted variable bias.

Indeed, Hess and Torney’s studies, which have explicitly measured political attitudes (see [92,93]), used themes and pictures to identify that children possessed independent political attitudes. More recently, Abendschön [57], Haug [62] and Van Deth et al. [60] focused on pre-school children, also using a novel set of pictures and other tasks, and found that children possessed specific political dispositions before their first year of school. Regarding children, when it is possible to measure the foundations of attitudes very early in life, attitudes are evident. This makes sense given that personality theorists included attitudes and political values as part of the measures used to assess personality [8,26,94].

It may also be possible that the time-period tested (10 years) is too long or too short for personality to have an influence on attitudes. The case of too short is not impossible, but appears highly improbable. If, for example, it were argued that we would need 20 years to identify a meaningful relationship between personality and attitudes, it would mean we should find little variability in attitudes or personality, but this was not the case. We observed substantial changes in personality and attitudes. In addition, it would also require a radical revision to personality theory, suggesting personality has almost no daily role in guiding individual preferences, behaviors, or selection into environments, but rather remains latent until some specified event or age.

What of the opposite? What if 10 years is too long a time-period, and the influence of personality on attitudes would be convoluted by other factors, due to the many changes that occur throughout a person’s life? While possible, if true, it would mean over the long run personality would have diminishing influence on attitudes, and personality only works in absence of other social factors. Such propositions, again, would challenge the very core of personality theory. In addition, we would expect to see substantially greater variability in attitudes relatively to personality traits, but this was not the case.

Perhaps it is useful to encapsulate these processes by considering the two mechanisms at the extremes of logical possibility. The first mechanism is a change associated with a single catalytic event resulting in a major shift in either personality or attitudes. If this type of change occurs, then the current model would only capture the causal implications if the event occurred within the measurement interval. If the event occurred before the initial measurement occasion or after the final measurement occasion, the model cannot incorporate the causal effect. While this is possible, is theoretically highly improbable and inconsistent with our findings. Our findings rely on two large populations, using a variety of measures, where the age of the participants varied both within and between samples that capture both early and mid to later life cohorts.

The second mechanism, proposes that near continuous, subtle, glacial changes occur throughout the life span. This mechanism is proffered by the architects of modern personality theories and is consistent with the overall literature on personality and behaviors at large [41,90]. In this case, minor, cumulative changes to both personality and attitudes would occur across a long period. The longer the time between measurements, the larger the possibility of change, and the greater possibility of observing any causal effects that may be present. This type of causality would be highly detectable in our models, if it were present. Accordingly, in the current analysis substantial within-person changes in both personality traits and political attitudes were observed, indicating that the time lag between measurement occasions was sufficient to observe these changes.

Thus, our interpretation of the evidence based on the current data, is that the commonality between personality traits and political attitudes is a function of a relationship that emerges quite early in the developmental process. This conclusion is highly consistent with previous studies [5,22,23] that find that the primary source of the relationship between personality traits and political attitudes is a function of shared genetic variance. Accordingly, this predicts if the correlation between political preferences and personality traits were the expression of the same underlying genetic variance, the relationship between personality traits and political attitudes at subsequent measurement occasions would be insignificant if the autoregressive components are accounted for within each domain, which is precisely what we find.

### Implications

This study has several implications for the theoretical development of the personality and attitudes literature. Specifically, the view that personality traits causally influence political attitudes is overstated at a minimum. If personality was truly causing people to hold political attitudes then (1) personality traits would likely be more stable than attitudes, which is not observed, (2) the correlation between personality traits and attitudes would increase over time, but it actually decreases, and (3) changes in personality over time should strongly coincide with changes in political attitudes, yet this is not the case. Over a ten-year span, we observe at best, only a trivial relationship in our analyses.

Accordingly, the shared properties of personality and attitudes are most likely a function of an unobserved trait that drives the development of both personality traits and political attitudes measured in two separate domains. This interpretation makes sense from both an empirical and a theoretical perspective. In terms of confirmatory factor analysis, many of the personality traits are quite political in nature, as certain facets of personality were intended to measure political attitudes and therefore, political attitudes should be correlated with personality traits by definition [10,11]. This is the perspective that McCrae and Costa took when including the politically charged values facet within the Openness to Experience Dimension of the NEO-PI-R [9]. Openness is often described as “a readiness to re-examine traditional social, religious, and political values” [95] or to capture moral beliefs and include attitudinal content that resembles political conservatism [96,97].

This possibility is also consistent with an underlying genetic factor that mutually influences both attitudes and personality traits. In this way, the components that covary are manifestations of the same construct that are measured and expressed in different domains. It also re-invigorates developmental approaches to the topic [98–102]. As Sullivan et al. [7] argue:

An underlying premise of this approach might be that the development of political thought and behavior is an integral part of general human development and that an understanding of the former cannot be divorced entirely from an understanding of the latter. Political behavior and thought are, quite simply, part and parcel of all human behavior and thought.

In conclusion, we believe a better understanding of the relationship between personality traits and political attitudes is only possible by considering attitudes independent and meaningful constructs, and as part of one’s disposition which guides behavior, as much as their personality. This view remains consistent with the development of personality traits. Recognizing the data are the most comprehensive to date, but remain fallible, our findings, in combination with the accumulation of previous studies [23], suggest that a purely causal relationship between personality traits and political attitudes does not exist. The results presented support the need to revise or reconcile recent research with long standing theoretical assumptions.

## Supporting Information

S1 File Items Used to Define Openness. (DOCX)

S2 File Examples of Political values Included in Measures of Personality. (DOCX)

S3 File Cross-Lagged Correlation Analysis: Correlations between the latent traits for the Adult Cohort. (DOCX)

S4 File Cross-Lagged Differential for the Adult Cohort. (DOCX)

S5 File Correlations between the latent traits for the Adolescent Cohort. (DOCX)
