# Correction: Political Attitudes Develop Independently of Personality Traits

**DOI:** 10.1371/journal.pone.0134072

**Published:** 2015-07-24

**Authors:** Peter K. Hatemi, Brad Verhulst

There is an error in the third sentence of the fourth paragraph in “The Nature of Personality Traits and Political Attitudes” section of the Introduction. The correct sentence is: For the FFM and Eysenck’s personality traits, correlations between Openness and social attitudes, Neuroticism and economic attitudes, Conscientiousness and social attitudes, the P-Scale and military/defense attitudes, and Social Desirability and social attitudes are the most consistently found.

There is an error in the eighth sentence of the first paragraph in the Measures section of the Materials and Methods. The correct sentence is: Attitude factors were coded so that higher values reflect more liberal attitude positions.
