# Supplementary material for: Political Attitudes Develop Independently of Personality Traits
Source: PLoS One. 2015 Mar 3;10(3):e0118106. doi: 10.1371/journal.pone.0118106 (PMC4347987; doi:10.1371/journal.pone.0118106)
Supplement: S1 File — (DOCX) [file pone.0118106.s001.docx]

**S1: Items Used to Define Openness**

| **Openness** |
| --- |
| Aesthetically reactive |
| Values intellectual matters |
| Wide range of interests |
| Rebellious, non-conforming |
| -vs- |
| Sex-role stereotyped behavior |
| Favors **conservative** Values |
| Uncomfortable with complexities |
| Judges in conventional terms |

Table adapted from Table 3 in Costa Jr, Paul T., and Robert R. McCrae. 1992. "Four Ways Five Factors Are Basic." *Personality and Individual Differences* 13: 653-65
