# Supplementary material for: Political Attitudes Develop Independently of Personality Traits
Source: PLoS One. 2015 Mar 3;10(3):e0118106. doi: 10.1371/journal.pone.0118106 (PMC4347987; doi:10.1371/journal.pone.0118106)
Supplement: S2 File — (DOCX) [file pone.0118106.s002.docx]

**S2: Examples of Political values Included in Measures of Personality**

| **Personality Measure** | **Dimension** | **Attitude Measure** |
| --- | --- | --- |
| We can never do too much for the poor and elderly | Agreeableness | welfare attitudes |
| Human need should always take priority over economic considerations | Agreeableness | economic policy attitudes |
| I would rather be known as “merciful” than as “just.” | Agreeableness | punitive attitudes |
| I don't take civic duties like voting very seriously | Conscientiousness | Voter turnout |
| I believe that laws and social policies should change to reflect the needs of a changing world | Openness | gay rights/euthanasia |
| I believe that the "new morality" of permissiveness is no morality at all. | Openness | gay rights |
| I consider myself broad-minded and tolerant of other people’s lifestyles. | Openness | social liberalism |
| I believe that different ideas of right and wrong that people in other societies have may be valid for them. | Openness | Conservatism |
