# Supplementary material for: Political Attitudes Develop Independently of Personality Traits
Source: PLoS One. 2015 Mar 3;10(3):e0118106. doi: 10.1371/journal.pone.0118106 (PMC4347987; doi:10.1371/journal.pone.0118106)
Supplement: S3 File — (DOCX) [file pone.0118106.s003.docx]

**S3. Cross-Lagged Correlation Analysis: Correlations between the latent traits for the Adult Cohort**

|  | P_80_ | SD_80_ | Religion_80_ | Soc Ideo_80_ | P_90_ | SD_90_ | Religion_90_ | Soc Ideo_90_ |
| --- | --- | --- | --- | --- | --- | --- | --- | --- |
| P_80_ | **1.000** |  |  |  |  |  |  |  |
| SD_80_ | **-0.552** | **1.000** |  |  |  |  |  |  |
| Religion_80_ | **0.518** | **-0.261** | **1.000** |  |  |  |  |  |
| Soc Ideo_80_ | **0.257** | **-0.193** | **0.653** | **1.000** |  |  |  |  |
| P_90_ | *0.670* | -0.279 | 0.308 | 0.206 | **1.000** |  |  |  |
| SD_90_ | -0.322 | *0.691* | -0.095 | -0.125 | **-0.457** | **1.000** |  |  |
| Religion_90_ | 0.311 | -0.121 | *0.757* | 0.585 | **0.399** | **-0.124** | **1.000** |  |
| Soc Ideo_90_ | 0.195 | -0.154 | 0.597 | *0.814* | **0.167** | **-0.151** | **0.670** | **1.000** |

The essential test of spuriousness is derived from cross-lagged differential in the simple formula presented in equation 1 (Kenny, 1975):ρ_x1y2_ – ρ_x2y1_ . Where ρ_x1y2_ is the correlation between Trait A in 1980 and Trait B in 1990 and ρ_x2y1_ is the correlation between Trait B in 1980 and Trait A in 1990. If the cross-lagged differential is positive, then it is generally concluded that X causes Y, while if the differential is negative then the conclusion is that Y causes X assuming that the correlations are all positive, or vice versa if the correlations are negative. The full longitudinal correlation matrix between P, Social Desirability, Religious and Social Ideology attitudes for the adult sample are presented. The correlation matrix can be further broken down into several sub-matrices corresponding. The first two sub-matrices are the two lower triangular matrices consisting of synchronous correlations, one from the first wave and one from the second wave. For ease of presentation, these matrices are presented in **Boldface Type**. The third sub-matrix is a full matrix consisting of autocorrelations on the diagonal and cross-lagged correlations on the off diagonal. For identification purposes, the autocorrelations are presented in *italic type* while the cross-lagged correlations are presented in standard type.The synchronous correlations are presented in bold type, the autocorrelations are presented in italics and the cross-lagged correlations are presented in normal type. All of the correlations in the model are significant beyond the .001 level.
