# Supplementary material for: Political Attitudes Develop Independently of Personality Traits
Source: PLoS One. 2015 Mar 3;10(3):e0118106. doi: 10.1371/journal.pone.0118106 (PMC4347987; doi:10.1371/journal.pone.0118106)
Supplement: S4 File — (DOCX) [file pone.0118106.s004.docx]

**S4: Cross-Lagged Differential for the Adult Cohort**

| r_(x,y)_ | ρ_x1y2_ | ρ_x2y1_ | Δ |
| --- | --- | --- | --- |
| r_(P,SD)_ | -0.322 | -0.279 | -0.043 |
| r_(P,Religion)_ | 0.311 | 0.308 | 0.003 |
| r_(P,Soc Ideo)_ | 0.195 | 0.206 | -0.011 |
| r_(SD, Religion)_ | -0.121 | -0.095 | -0.026 |
| r_(SD, Soc Ideo)_ | -0.154 | -0.125 | -0.029 |
| r_(Religion, Soc Ideo)_ | 0.597 | 0.585 | 0.012 |

The cross-lagged differentials are presented. The first variable in the parentheses corresponds to the x variable and the second variable in the parentheses corresponds with the y variable. None of the cross-lagged differentials are significant at the conventional level of .05. As can be seen, none of the cross-lagged differentials are statistically distinct from equality (*p’s* > .05). According to this analysis, we are forced to conclude that the relationship between the personality traits and the attitude dimensions in the adult sample are spurious.
