# Supplementary material for: Political Attitudes Develop Independently of Personality Traits
Source: PLoS One. 2015 Mar 3;10(3):e0118106. doi: 10.1371/journal.pone.0118106 (PMC4347987; doi:10.1371/journal.pone.0118106)
Supplement: S5 File — (DOCX) [file pone.0118106.s005.docx]

**S5: Correlations between the latent traits for the Adolescent Cohort**

|  | Openness to Experience (1998) | Values  (1998) | Openness to Experience (2007) | Social Attitudes  (2007) |
| --- | --- | --- | --- | --- |
| Openness to Experience (1998) | **1.000** |  |  |  |
| Values  (1998) | **-0.500** | **1.000** |  |  |
| Openness to Experience (2007) | *0.533* | -0.170 | **1.000** |  |
| Social Ideology  (2007) | -0.207 | *0.517* | **-0.132** | **1.000** |

The synchronous correlations are presented in bold type, the autocorrelations are presented in italics and the cross-lagged correlations are presented in normal type. All of the correlations in the model are significant beyond the .01 level. Again, the cross-lagged differential was not statistically distinct from equality suggesting that the relationship between openness and social attitudes is driven by an additional unmeasured factor.
